# A new modified obstetric early warning score for prognostication of severe maternal morbidity

**DOI:** 10.1186/s12884-022-05216-7

**Published:** 2022-12-05

**Authors:** Yonghui Xu, Sha Zhu, Hao Song, Xiaoyuan Lian, Maoni Zeng, Ji He, Lijuan Shu, XingSheng Xue, Fei Xiao

**Affiliations:** 1 Department of Obstetrics and Gynecology Intensive Care Unit, West China Women’s and Children’s Hospital, Sichuan University, No. 20, Section 3, Renmin South Road, Chengdu, China; 2 Key Laboratory of Birth Defects and Related Diseases of Women and Children, Sichuan University, Ministry of Education, Chengdu, China

**Keywords:** Modified obstetric early warning score (MOEWS), APACHE II score, Maternal morbidity, Maternal mortality, Intensive care unit (ICU)

## Abstract

**Background:**

Maternal mortality is still a major challenge for health systems, while severe maternal complications are the primary causes of maternal death. Our study aimed to determine whether severe maternal morbidity is effectively predicted by a newly proposed Modified Obstetric Early Warning Score (MOEWS) in the setting of an obstetric intensive care unit (ICU).

**Methods:**

A retrospective study of pregnant women admitted in the ICU from August 2019 to August 2020 was conducted. MOEWS was calculated 24 h before and 24 h after admission in the ICU, and the highest score was taken as the final value. For women directly admitted from the emergency department, the worst value before admission was collected. The aggregate performance of MOEWS in predicting critical illness in pregnant women was evaluated and finally compared with that of the Acute Physiology and Chronic Health Evaluation II (APACHE II) score.

**Results:**

A total of 352 pregnant women were enrolled; 290 women (82.4%) with severe maternal morbidity were identified and two of them died (0.6%). The MOEWSs of women with serious obstetric complications were significantly higher than those of women without serious obstetric complications [8(6, 10) vs. 4(2, 4.25), z = -10.347, *P* < 0.001]. MOEWSs of 24 h after ICU admission had higher sensitivity, specificity and AUROC than MOEWSs of 24 h before ICU admission. When combining the two MOEWSs, sensitivity of MOEWS was 99.3% (95% CI: 98–100), specificity 75.8% (95% CI: 63–86), positive predictive value (PPV) 95.1% (95% CI: 92–97) and negative predictive value (NPV) 95.9% (95% CI: 86–100). The areas under the receiver operator characteristic (ROC) curves of MOEWS were 0.92 (95% CI: 0.88–0.96) and 0.70 (95% CI: 0.63–0.76) of the APACHE II score.

**Conclusion:**

The newly proposed MOEWS has an excellent ability to identify critically ill women early and is more effective than APACHE II. It will be a valuable tool for discriminating severe maternal morbidity and ultimately improve maternal health.

**Supplementary Information:**

The online version contains supplementary material available at 10.1186/s12884-022-05216-7.

## Background

Maternal mortality is still a major concern for health systems worldwide [1]. Significant improvements in maternal health have been made to date and the maternal mortality ratio(MMR) has obviously decreased. It remains a challenge, however, among vulnerable women, especially in low income countries [2]. Serious obstetric complications are recognized as primary contributors to maternal death [3, 4]. The World Health Organization (WHO) recommends that maternal safety surveillance should focus not only on maternal mortality but also on severe maternal morbidity. Valid identification of women with high-risk pregnancies will help to improve maternal health [5].

It is known that obstetric haemorrhage, maternal sepsis, hypertensive disorders of pregnancy, abortion, cardiovascular diseases, amniotic fluid embolism and pulmonary embolism are the most common causes of maternal death [1, 6]. Maternal morbidity and mortality are often preventable. Timely identification of obstetric complications with appropriate management can reduce the burden of maternal mortality. Women with potentially life-threatening complications are not easily identified, however [7, 8]. APACHE II score is a widely used prognostic scoring system in nonpregnant populations [9, 10]. Since pregnancy is accompanied by a series of physiological changes, the APACHE II score may not entirely be applicable to women in obstetric ICU settings.

At present, multiple modified obstetric early warning scores (EWS) are widely used for early identification of critically ill women with obstetric complications. The Modified Early Obstetric Warning Score (MEOWS) [5, 11, 12], Maternal Early Recognition Criteria (MERC) [13, 14], Modified Early Warning System (MEWS) [2, 15], Maternal Early Warning Trigger (MEWT) [16, 17], Maternal Early Obstetric Warning System (MEOWS chart) [18, 19], Irish Maternity Early Warning System (IMEWS) [20, 21] and ICNARC Obstetric Early Warning Score (OEWS) [7, 22] are the most common. In 2020, ICNARC OEWS was first compared with the APACHE II score in India [22]. In the same year, a new MEOWS was designed in Rwanda, but sensitivity and PPV were low [23]. Main advantage of all scoring systems is that they are based on the physiological changes during pregnancy and do not require any laboratory tests. Most predictive models have been verified to recognize parturients at high risk of developing severe maternal morbidity. Nevertheless, there have been no universal standards for obstetric EWS until now, and the parameter settings of each scoring system vary, as do sensitivity and specificity. Therefore, it makes sense to propose a new modified obstetric early warning score.

Primary objective of this study was to validate this new MOEWS for the prediction of deteriorating maternal conditions. The capacity of discernment was assessed by area under receiver operator characteristic (ROC) curves, sensitivity, specificity and predictive value. Furthermore, we compared the predictive ability of this new MOEWS with that of the APACHE II score for severe obstetric complications.

## Methods

### Aim

To determine the feasibility of implementing the MOEWS tool in the setting of an obstetric ICU in the department of Obstetrics and Gynecology, West China Women’s and Children’s Hospital of Sichuan University. This is one of the first national key clinical specialty constructions and is the treatment and referral center of pregnant women with severe obstetric complications in western China, where about 19,000 births occur annually.

### Study design and study population

This is a retrospective study of a total of 19,438 births occurring in our hospital from August 2019 to August 2020. There were 378 women with obstetric complications admitted to the obstetric ICU. They were either pregnant or women within 42 days after childbirth and 352 women with an ICU stay of at least 24 h.

### Data collection

Since many women were in a critical condition before ICU admission, we assessed MOEWS 24 h before and 24 h after admission to ICU and the highest score was taken as the final value. For women directly admitted from the emergency department, the worst value before admission was collected. In addition, APACHE II score was calculated within 24 h of ICU admission. For calculation of the score, the most abnormal reading of each clinical and laboratory parameter was taken. The predicted maternal mortality of APACHE II was also calculated by medical software. Finally, MOEWS scores and APACHE II scores were compared to distinct the prediction ability of MOEWS and APACHE II.

### Subject evaluation

Parameters and their numerical values used for the calculation of MOEWS are shown in Table 1. Measurements of temperature, respiratory rate, peripheral oxygen saturation, method of oxygen therapy (nasal catheters, mask, non-invasive or invasive ventilator), heart rate, blood pressure and consciousness level (alert, responsive to sound or pain and unresponsive) were documented. Regarding the APACHE II score, all laboratory parameters were obtained through venous blood sampling. The partial pressure of oxygen (PaO2) in arterial blood (in mmHg) was recorded from the arterial blood gas (ABG) analysis.

The modified World Health Organization (mWHO) classification of maternal cardiovascular risk was used to assess the maternal risk of cardiac complications [24]. In this study, women with cardiovascular diseases whose mWHO risk classification was III or IV were identified as high-risk. For pregnant women with heart disease, bedside echocardiography was used to assess cardiac structure and function in emergencies.

Vital signs indicating cardiac disease and postpartum haemorrhage, however, were not significantly abnormal in the early stages because of physiological changes in pregnancy. For women with postpartum haemorrhage, much more blood loss has to occur as compared to non-pregnant women before significant deterioration of vital signs. Consequently, three points were added to MOEWS in women confirmed to have high-risk cardiovascular disease or severe postpartum haemorrhage.

Study endpoint was severe maternal morbidity (definitions shown in Table 2). Clinical characteristics were collected, including age and gestational age. Secondary outcomes were length of ICU stay, requirement for ventilation, vasopressors, intravenous antihypertensive drugs, transfusion, hysterectomy, arterial embolization, haemodialysis and extracorporeal membrane oxygenation (ECMO).

**Table 1:** Cut-off limits of individual parameters of MOEWS score

Score	3	2	1	0	1	2	3
Temperature (°C)	≤35		<36	36-37.4	37.5-38	38.1-38.9	≥39
Respiratory rate (beats/min)	<10		10-11	12-20		21-29	≥30
SPO2 (%)	≤90	91-93	94-95	≥96			
Oxygen therapy	Mask or above	Nasal catheter		Room air			
Heart rate (beats/min)	<50	50-59		60-99	100-109	110-129	≥130
Systolic blood pressure (mmHg)	<90		90-99	100-139	140-149	150-159	≥160
Diastolic blood pressure (mmHg)			≤45	46-89	90-99	100-109	≥110
Consciousness level				Alert			Not alert

For women with severe postpartum haemorrhage or high-risk cardiovascular disease (mWHO III or IV) (Supplementary Table 1), 3 points were added to the total score

Recommended clinical reaction: The critical care system changes colour based on the score of each parameter to alert the ICU team to initiate clinical responses, with scores of 3 turning red and 2 turning yellow. When the total MOEWS ≤ 2, the current plan is maintained; when MOEWS = 3–4, the observations are repeated; when MOEWS ≥ 5 or a single parameter score is 3, the woman is admitted to the ICU. *The definition of severe postpartum haemorrhage was shown in Table 2 and the details of mWHO classification of maternal cardiovascular risk was shown in a Supplementary Table 1.

**Table 2:** Diagnostic criteria of severe maternal morbidity

Severe maternal morbidity	Diagnostic criteria
Pre-eclampsia [19]	SBP ≥ 160 mmHg, or DBP ≥ 110 mmHg plus proteinuria ≥ 0.3 g. day-1 (+ 2 dipstick) or hypertension (≥ 140/90 mmHg) and proteinuria with at least one of the following: headache; visual disturbance; epigastric pain; clonus; platelet count ≤ 100 0.109 -1); AST > 50 iu.l-1; Crvf > 100 umol.l -1; or CrCl < 80.8 ml.min-1. Severe hypertension requiring treatment with intravenous antihypertensive agents.
Eclampsia [25]	Severe preeclampsia characterized by sudden onset of generalized tonic-clonic seizures
Severe postpartum haemorrhage [14, 26]	Documented estimated blood loss ≥ 2000 ml, need for blood transfusion of at least 3 U, or hysterectomy, with or without radiological embolization of uterine arteries
Suspected sepsis [19]	Clinically suspected focus of infection ± positive laboratory culture, treated with antibiotics (excluding commensals and antibiotic prophylaxis)
Shock [18]	Persistent severe hypotension defined as systolic blood pressure < 90 mmHg for 60 min or decreased by 40 mmHg with increased pulse rate
Pulmonary oedema [19]	Breathlessness, crepitations requiring diuretics
Thromboembolism [12]	CTPA confirmed pulmonary embolism, venous thrombosis in the pelvic region, deep venous thrombosis in the extremities or sinus thrombosis
Diabetic ketoacidosis [19]	Hyperglycaemia, metabolic acidosis, ketones in urine
Intracranial tumour [19]	CT ⁄ MRI confirmed
Status epilepticus [19]	History of epilepsy, prolonged multiple seizures
Other serious medical conditions [19, 27–29]	Acute pancreatitis, liver failure, acute fatty liver of pregnancy, acute appendicitis or other critical illness

*SBP *Systolic blood pressure, *DBP *Diastolic blood pressure, *AST *Aspartate aminotransferase, *Cr *Creatinine, *CrCL *Creatinine clearance, *CTPA *Computed tomography pulmonary angiogram, *MRI *Magnetic resonance imaging

### Statistical analysis

Results were tabulated and subjected to statistical analysis using Statistical Package for Social Sciences (SPSS) version 25.0. ROC curves were used to determine the area under the curve of various scores for predicting severe morbidity. Sensitivity, specificity, positive predictive values (PPVs) and negative predictive values (NPVs) were calculated. For all statistical tests, we regarded a value of p < 0.05 as statistically significant.

## Results

A total of 378 pregnant women were admitted to ICU, 26 with an ICU stay of less than 24 h were excluded, leaving 352 women in the study. A total of 290 women (82.4%) experienced serious complications and two of them died (0.6%). One of these two died from pulmonary embolism and the other from severe pulmonary hypertension. The most frequent causes of ICU admission were hypertensive disorders of pregnancy (108/352; 30.7%), followed by cardiovascular disease (94/352; 26.7%), obstetric haemorrhage (61/352; 17.3%) and suspected sepsis (22/352; 6.3%) (Table 3).

**Table 3:** Distribution of maternal morbidity

Indications for admission to ICU	Frequency (***n*** = 352)	Percentage (%)
Hypertensive disorders of pregnancy	108	30.7
Cardiovascular disease	94	26.7
Obstetric haemorrhage	61	17.3
Suspected sepsis	22	6.3
Acute pulmonary edema	14	4.0
Pulmonary embolus	6	1.7
Liver disease (liver failure, severe hepatitis, acute fatty liver of pregnancy, hepatitis B cirrhosis)	6	1.7
Systemic lupus erythematosus (SLE)	6	1.7
Acute pancreatitis	2	0.6
Acute appendicitis	2	0.6
Anaphylaxis	3	0.9
Gastrointestinal haemorrhage	2	0.6
Malignancy	5	1.4
Diabetic ketoacidosis	1	0.3
Hyperthyroidism crisis	2	0.6
Other medical disorders	26	7.4
Neurological diseases	5	1.4
Respiratory diseases	3	0.9
Blood disease	4	1.1
Deep vein thrombosis (DVT)	2	0.6
Renal transplantation	1	0.3
Others^a^	11	3.1

^a^Include the chronic renal failure, cavernous transformation of the portal vein, unidentified hypoxia during operation and intraoperative arrhythmia

**Fig. 1:**
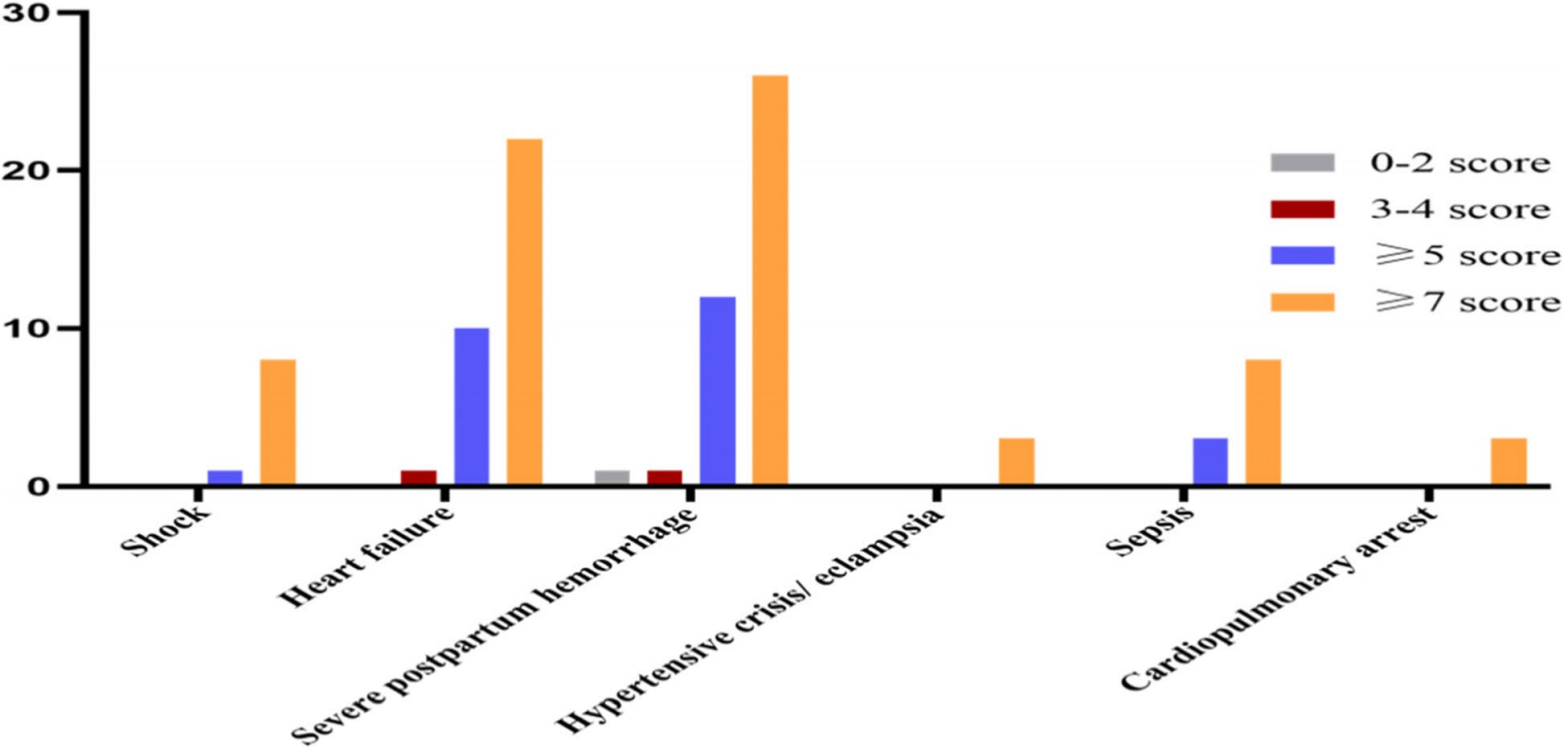
Relationship between MOEWS and serious complications

**Fig. 2:**
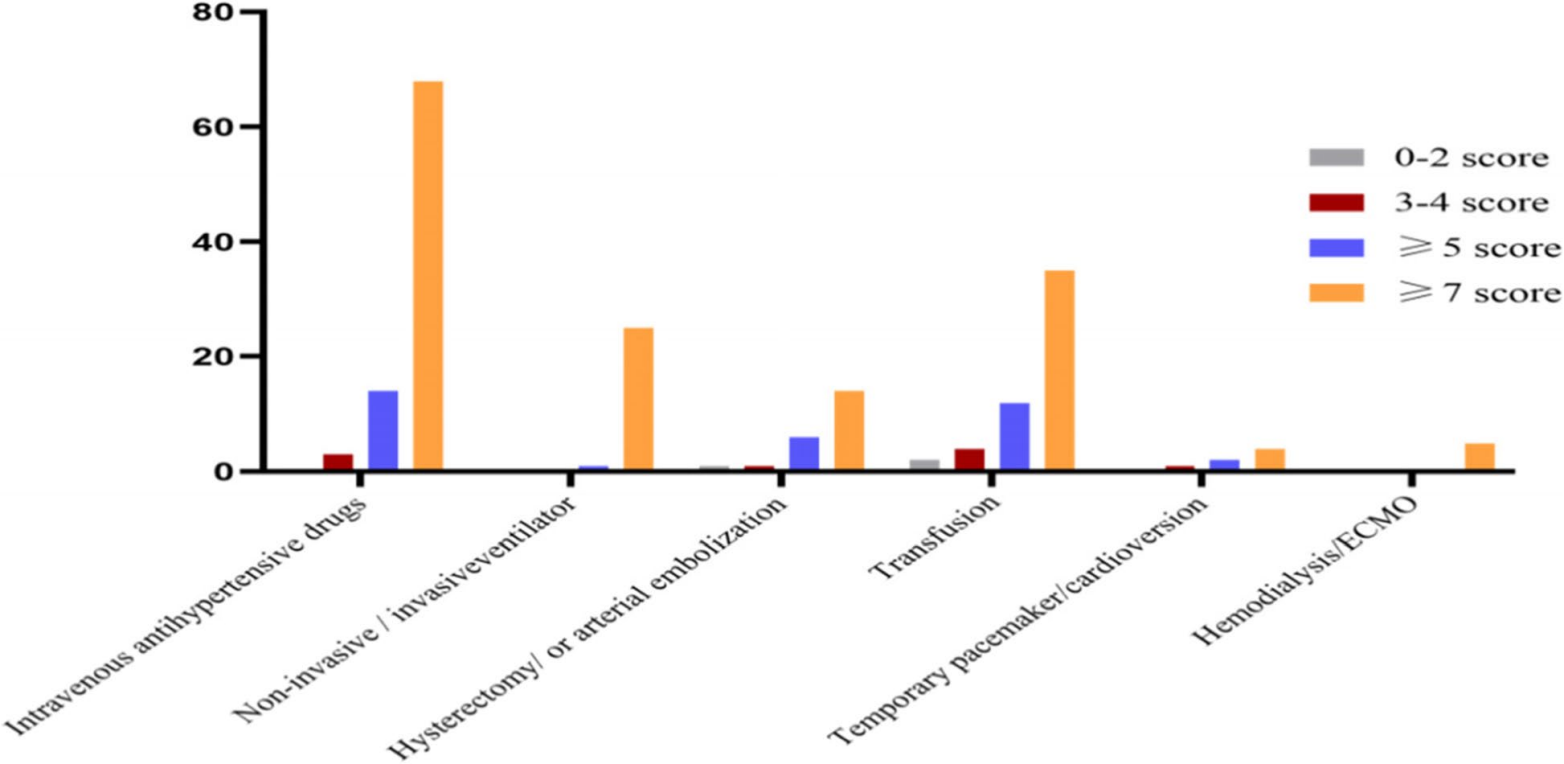
Correlation between MOEWS and life support interventions

Good risk stratification ability was demonstrated by MOEWS. Shock occurred in nine pregnant women, heart failure in 33 and sepsis in nine. Meanwhile, 85 women were treated with intravenous antihypertensive drugs, two of whom developed eclampsia and one experienced a hypertensive crisis. Forty women suffered from severe postpartum haemorrhage, 22 of whom underwent hysterectomy or arterial embolization. Seven of those with serious heart arrhythmia got a temporary pacemaker or external direct cardioversion. Noninvasive or invasive artificial ventilation and bloodtransfusion were mostly used in women with high MOEWSs, as were those with haemodialysis and ECMO, especially in the MOEWS ≥ 7 group (Figs. 1 and 2).

MOEWS was correlated with the length of ICU stay and gestational age. Median MOEWS was 7 (5, 9.75) and 4 (2, 6) with APACHE II. In the high-risk group (score ≥ 5), median APACHE II score [4 (2, 6)] and length of ICU stay [4(2, 4)] were higher than those in the low-risk (score 0–2) and moderate-risk (score of 3–4) groups, while gestational age [34 ± 2.5 (31 ± 1, 36 ± 4)] was shorter (*p* < 0.001). No statistically significant differences, however, were found between the low- and moderate-risk groups. Median age was not statistically significantly different among all groups (*p* = 0.628) (Table 4).

**Table 4:** Correlation of MOEWS and clinical characteristics

MOEWS score	Total	Critically ill	APACHE II score	Length of ICU stay (days)	Age (years)	Gestational age (weeks)
0–2	16	2	2(0.5, 3)	2(1, 2)	31.5(29.3, 36.0)	38 ± 1.5(36 ± 0, 38 ± 4)
3–4	47	14	3(2, 5)	2(1, 2)	31.0(27.0, 34.0)	36 ± 2.0(33 ± 1, 38 ± 5)
≥ 5	289	274	4(2, 6)*	4(2, 4) *	32.0(28.0, 35.0)	34 ± 2.5(31 ± 1, 36 ± 4) *
H value			18.068	18.797	0.931	18.504 ± 1.667
*P* value			< 0. 001	< 0.001	0.628	< 0.001

All outcomes are shown with IQR (P25, P75); * indicates statistical significance, *P*<0.001

Median score of MOEWS within 24 h before and 24 h after ICU admission was 5 (3, 8) and 7 (5, 9), with 5.5 (3, 8) and 7 (5, 9) in critically ill women, while this was 2 (0, 3.25) and 3 (2, 4) (*p* < 0.001) in those without critical illness. Combining the two MOEWSs, median score was 7 (5, 9.75), with 8 (6, 10) in the critically ill group and 4 (3, 4.25) in those without critical illness (*p* < 0.001). Median score of APACHE II was 4 (2, 6), while it was 4 (2, 6) in those with critical illness and 2 (2, 4) in those without critical illness (*p* < 0.001) (Table 5).

**Table 5:** Comparison of MOEWS and APCHE-II score between critically ill and not critically ill women

Score	Median	Not critically ill (95% CI)	Critically ill (95% CI)	Z value	*P* value
MOEWS1	5 (3, 8)	2 (0, 3.25) (1.67–2.75)	5.5 (3,8) * (5.57–6.43)	-7.951	< 0.001
MOEWS2	7 (5,9)	3 (2,4) (2.91–3.7)	7 (5,9) * (7.23–7.96)	-10.177	< 0.001
MOEWS	7 (5, 9.75)	4 (3,4.25) (3.38–4.24)	8 (6,10) * (7.93–8.68)	-10.347	< 0.001
APACHE II	4 (2, 6)	2 (2,4) (2.25–3.26)	4 (2,6) * (4.47–5.43)	-4.926	< 0.001

MOEWS1is the score of 24 hours before ICU admission; MOEWS2 is the score of 24 hours after ICU admission; MOEWS means the highest score of MOEWS1 and MOEWS2; median scores with IQR (P25, P75); * indicates statistical significance, *P*<0.001

**Fig. 3:**
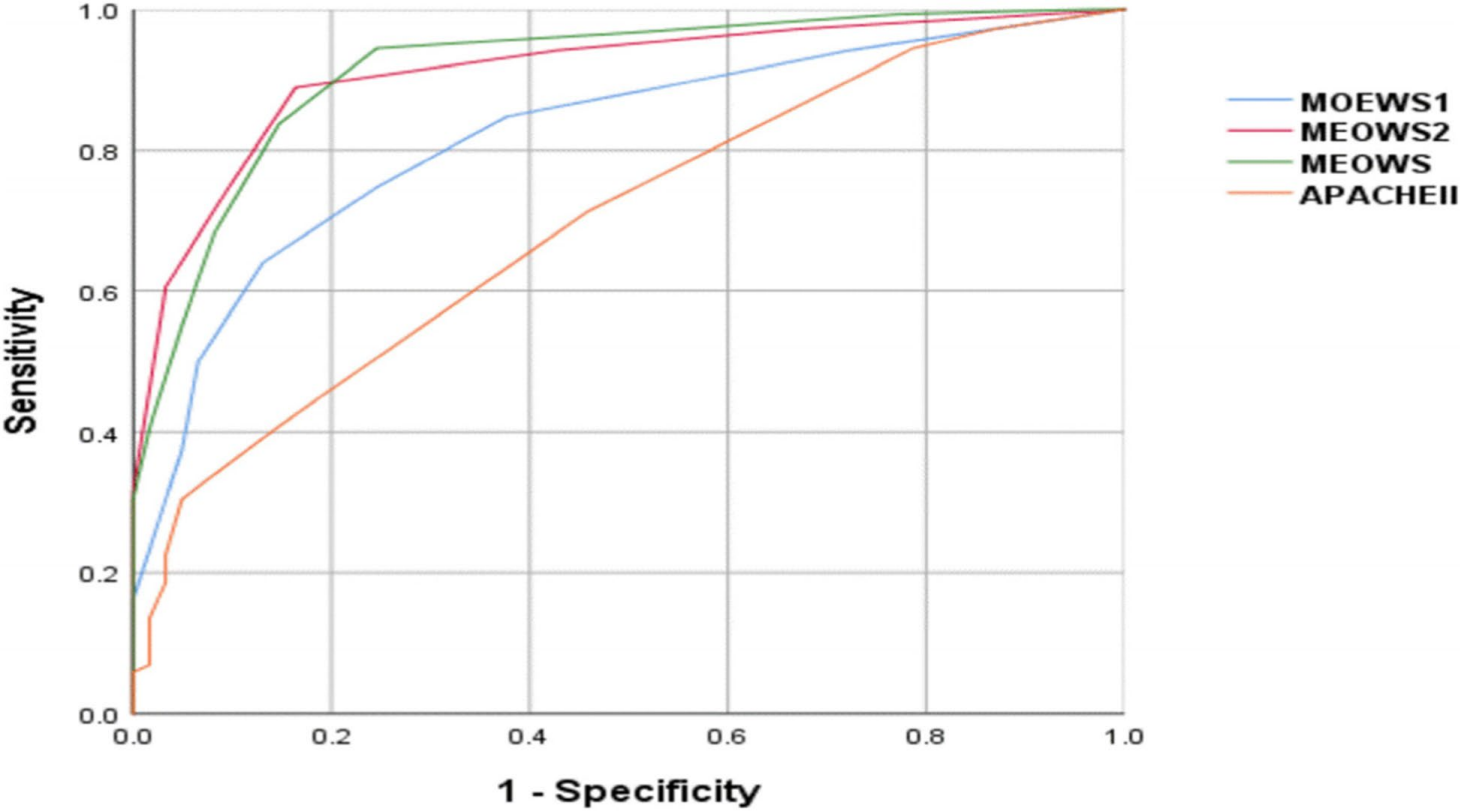
ROC curves of MOEWS and APACHE II score for prediction of maternal morbidity. MOEWS1 is score 24 h before ICU admission; MOEWS2 is score 24 h after ICU admission; MOEWS is the highest of MOEWS1 and MOEWS2

AUROC of the MOEWSs of 24 h before admission to ICU was 0.82 (95% CI: 0.77–0.87) and 0.91 (95% CI: 0.87–0.95) 24 h thereafter (Fig. 3). When the cut-off was > 4, all MOEWs test-indicators are given in Table 6. Meanwhile, AUROC of APACHE II was 0.70 (95% CI: 0.63–0.76) (Fig. 3). When the cut-off was ≥ 10, all Apache II test-indicators can be found in Table 6. The predicted maternal mortality of APACHE II was 8.4(95% CI: 8.77–25.89) (Table 6), higher than the observed maternal mortality (0.6%).

**Table 6:** Validity of the MOEWS and APACHE II score to predict maternal morbidity

Score	Cut-off	AUROC (95% CI)	Sensitivity (95% CI)	Specificity (95% CI)	PPV (95% CI)	NPV (95% CI)	Predicted maternal mortality (95% CI)
MOEWS1	>4	0.82 (0.77-0.87)	84.8% (80-89)	87.1% (76-94)	96.9% (94-99)	55.1% (45-65)	
MOEWS2	>4	0.91 (0.87-0.95)	96.9% (98-100)	83.9% (72-92)	96.6% (94-98)	85.2% (74-93)	
MOEWS	>4	0.92 (87.8-95.5)	99.3% (98-100)	75.8% (63-86)	95.1% (92-97)	95.9% (86-100)	
APACHE	≥	0.70*	10.3%	98.4%	96.8%	8.1%	8.4%
II	10	(0.63-0.76)	(7-14)	(91-100)	(83-100)	(15-24)	(8.77-25.89)
*P* value		0.001					

MOEWS1 is score 24 hours before ICU admission; MOEWS2 is score 24 hours after ICU admission; MOEWS is the highest score of MOEWS1 and MOEWS2; predicted maternal mortality of APACHE II score is shown with IQR (P25, P75); * indicates statistical significance, *P*<0.01

## Discussion

The identification of potentially critically ill women is an effective way to prevent the continuous deterioration of their condition and subsequently reduce maternal mortality. The implementation of the obstetric EWS has been found to be effective in predicting severe morbidity [12]. This is the first time to evaluate MOEWS both 24 h before and 24 h after admission to the ICU. Women with serious complications could be screened early to a great extent and an association between MOEWS and severe maternal morbidity could be demonstrated.

It was demonstrated in this study that the most common indication for admission to the obstetric ICU was preeclampsia. Cardiovascular diseases, obstetric haemorrhage and suspected sepsis followed in sequence. Shock, heart failure, severe postpartum haemorrhage, sepsis, hypertensive crisis and eclampsia were highly related to maternal mortality. Most critically ill women were accompanied by changes in vital signs. It was shown that women with preeclampsia, heart failure, shock and sepsis were easier to trigger by following vital signs only. Some conditions, however, could not be accurately judged only from vital signs and were prone to false-negative results, such as pregnancies with pulmonary hypertension, valvular heart disease and congenital heart disease. When combined with the mWHO classification of maternal cardiovascular risk, women with severe cardiovascular diseases could be easily identified at an earlier stage.

The risk of severe morbidity increased as MOEWS increased and correlated with increased length of ICU stay and lower gestational age. Delays in recognition, assessment and treatment are well recognized as significant contributors to maternal morbidity [15]. Thus, in order to identify women with a potential deterioration of their condition earlier, women with MOEWSs ≥ 5 or a single parameter score of 3 should be admitted to ICU for close monitoring.

Good discrimination of MOEWS was also verified by AUROC and other screenings indicators. MOEWS of 24 h after ICU admission was found to have higher sensitivity, specificity and AUROC than MOEWS of 24 h before ICU admission. The best test indicators resulted from a combination of the two MOEWSs. If MOEWSs can be promoted in general wards and emergency departments, potentially high-risk women may be identified to a greater extent in order to make closer monitoring and earlier treatment possible.

The AUROC of APACHE II scores was relatively low and tended to overpredict maternal deaths in this study, which was also observed in previous studies [9, 22, 30, 31]. This may be related to the pathophysiological changes in pregnancy [30]. APACHE II score thus should not be used to predict severe maternal morbidity in the setting of an obstetric ICU.

Generally, MOEWS is an effective predictor of severe maternal morbidity. It is simple and less time-consuming compared to APACHE II, as it is a bedside test only. For cardiovascular diseases, information for risk assessment can be obtained from the medical history or emergency bedside ultrasound.

Several limitations should be considered when interpreting the results of this study. Firstly, our study was only conducted in our hospital and may not be entirely applicable to other hospital settings. Secondly, it was a retrospective analysis with a relatively small sample size. Prospective studies are required to validate our conclusions.

## Conclusion

The MOEWS tool is feasible and acceptable in obstetric ICUs. Implementation of MOEWS may contribute to prevent deterioration of maternal morbidity by improving early identification of women with such conditions. Similarly, both quality of care and maternal health can be improved to a certain extent. Nonetheless, successful implementation of MOEWS needs further study to evaluate the efficiency of predicting severe maternal morbidity in other ICU settings.

## Supplementary Information

**Additional file 1: Supplementary Table 1. **Modified World Health Organization classification of maternal cardiovascular risk.

## Data Availability

The datasets used and/or analysed during the current study are available from the corresponding author on reasonable request.

## References

[1] Dzakpasu S, Deb-Rinker P, Arbour L, Darling EK, Kramer MS, Liu S (2020). Severe maternal morbidity surveillance: monitoring pregnant women at high risk for prolonged hospitalisation and death. Paediatr Perinat Epidemiol.

[2] Blumenthal E, Hooshvar N, McQuade M, McNulty J (2019). A validation study of maternal early warning Systems: a retrospective cohort study. Am J PePerinatol.

[3] Say L, Chou D, Gemmill A, Tunçalp Ö, Moller A-B, Daniels J (2014). Global causes of maternal death: a WHO systematic analysis. Lancet Glob Health.

[4] Schaap T, Bloemenkamp K, Deneux-Tharaux C, Knight M, Langhoff-Roos J, Sullivan E (2019). Defining definitions: a Delphi study to develop a core ou tcome set for conditions of severe maternal morbidity. BJOG.

[5] Ryan HM, Jones MA, Payne BA, Sharma S, Hutfield AM, Lee T (2017). Validating the performance of the modified early obstetric warning System Multivariable Model to predict maternal intensive care unit admission. J Obstet Gynaecol Canada.

[6] Zuckerwise LC, Lipkind HS (2017). Maternal early warning systems-towards reducing preventable maternal mortality and severe maternal morbidity through improved clinical surveillance and responsiveness. Semin Perinatol.

[7] Carle C, Alexander P, Columb M, Johal J (2013). Design and internal validation of an obstetric early warning score: secondary analysis of the Intensive Care National Audit and Research Centre Case Mix Programme database. Anaest hesia.

[8] Mhyre JM, D’Oria R, Hameed AB, Lappen JR, Holley SL, Hunter SK (2014). The maternal early warning criteria: a proposal from the national partnership for maternal safety. Obstet Gynecol.

[9] Lee JH, Hwang SY, Kim HR, Kim YW, Kang MJ, Cho KW (2017). Effectiveness of the sequential organ failure assessment, acute physiology and chronic health evaluation II, and simplified acute physiology score II prognostic scoring systems in paraquat-poisoned patients in the intensive care unit. Hum Exp Toxicol.

[10] Draper EA, Wagner DP, Zimmerman JE (1985). APACHE II: a severity of disease classification system. Crit Care Med.

[11] Mander R, Smith GD (2008). Saving mothers’ lives (formerly why mothers die): reviewing maternal deaths to make motherhood safer 2003–2005. Midwifery.

[12] Hoppu S, Hannola K, Mennander S, Huhtala H, Rissanen M, Tulensalo E (2020). Routine Bedside Use of Obstetric early warning system in the postnatal Ward to identify maternal morbidity among high-risk women. J Patient Saf.

[13] Klumpner TT, Kountanis JA, Langen ES, Smith RD, Tremper KK (2018). Use of a novel electronic maternal surveillance system to generate automated alerts on the labor and delivery unit. BMC Anesthesiol.

[14] Klumpner TT, Kountanis JA, Meyer SR, Ortwine J, Bauer ME, Carver A (2020). Use of a Novel Electronic maternal surveillance system and the maternal early warning criteria to detect severe Postpartum Hemorrhage. Anesth Analg.

[15] Friedman AM, Campbell ML, Kline CR, Wiesner S, D’Alton ME, Shields LE (2018). Implementing Obstetric early warning Systems. AJP Rep.

[16] Blumenthal EA, Hooshvar N, Tancioco V, Newman R, Senderoff D, McNulty J (2021). Implementation and evaluation of an electronic maternal early warning trigger Tool to reduce maternal morbidity. Am J Perinatol.

[17] Shields LE, Wiesner S, Klein C, Pelletreau B, Hedriana HL (2016). Use of Maternal Early Warning Trigger tool reduces maternal morbidity. Am J Obstet Gynecol..

[18] Singh A, Guleria K, Vaid NB, Jain S (2016). Evaluation of maternal early obstetric warning system (MEOWS chart) as a predictor of obstetric morbidity: a prospective observational study. Eur J Obstet Gynecol Reprod Biol.

[19] Singh S, McGlennan A, England A, Simons R (2012). A validation study of the CEMACH recommended modified early obstetric warning system (MEOWS). Anaesthesia.

[20] Maguire PJ, O’Higgins A, Power K, Turner MJ (2014). The irish maternity early warning system (IMEWS). Ir Med J.

[21] Nair S, Spring A, Dockrell L, Mac Colgain S (2020). Irish maternal early warning score. Ir J Med Sci.

[22] Khergade M, Suri J, Bharti R, Pandey D, Bachani S, Mittal P (2020). Obstetric early warning score for prognostication of critically ill obstetric patient. Indian J crit care Med peer-reviewed. Off Publ Indian Soc Crit Care Med.

[23] Tuyishime E, Ingabire H, Mvukiyehe JP, Durieux M, Twagirumugabe T (2020). Implementing the risk identification (RI) and modified early obstetric warning signs (MEOWS) tool in district hospitals in Rwanda: a cross-sectional study. BMC Pregnancy Childbirth.

[24] Regitz-Zagrosek V, Roos-Hesselink JW, Bauersachs J, Blomstrom-Lundqvist C, Cifkova R, De Bonis M (2019). 2018 ESC Guidelines for the management of cardiovascular diseases during pregnancy. Kardiol Pol.

[25] Clinical Guidelines - Queensland Health Q. Maternity and Neonatal Clinical Guideline. Queensl Heal. 2017;1–39. http://www.health.qld.gov.au/qcg%0Awww.health.qld.gov.au/qcg%0Awww.health.qld.gov.au/qcg. Accessed June, 2020.

[26] Henriquez DDCA, Gillissen A, Smith SM, Cramer RA, van den Akker T, Zwart JJ (2019). Clinical characteristics of women captured by extending the definition of severe postpartum haemorrhage with “refractoriness to treatment”: a cohort study. BMC Pregnancy Childbirth.

[27] Cruciat G, Nemeti G, Goidescu I, Anitan S, Florian A (2020). Hypertriglyceridemia triggered acute pancreatitis in pregnancy - diagnostic approach, management and follow-up care. Lipids Health Dis.

[28] Segev L, Segev Y, Rayman S, Nissan A, Sadot E (2017). Acute Appendicitis during pregnancy: different from the nonpregnant state?. World J Surg.

[29] Lim E, Mouyis M, MacKillop L (2021). Liver diseases in pregnancy. Clin Med (Northfield Il).

[30] Suri J, Kumar R, Gupta A, Mittal P, Suri JC (2020). A prospective study of clinical characteristics and interventions required in critically ill obstetric patients. Indian J Crit Care Med.

[31] Ryan HM, Sharma S, Magee LA, Ansermino JM, MacDonell K, Payne BA (2016). The usefulness of the APACHE II score in obstetric critical care: a structured review. J Obstet Gynaecol Canada.

