# Supplementary material for: A new modified obstetric early warning score for prognostication of severe maternal morbidity
Source: BMC Pregnancy Childbirth. 2022 Dec 5;22:901. doi: 10.1186/s12884-022-05216-7 (PMC9720996; doi:10.1186/s12884-022-05216-7)

Supplementary table1: Modified World Health Organization classification of maternal cardiovascular risk.


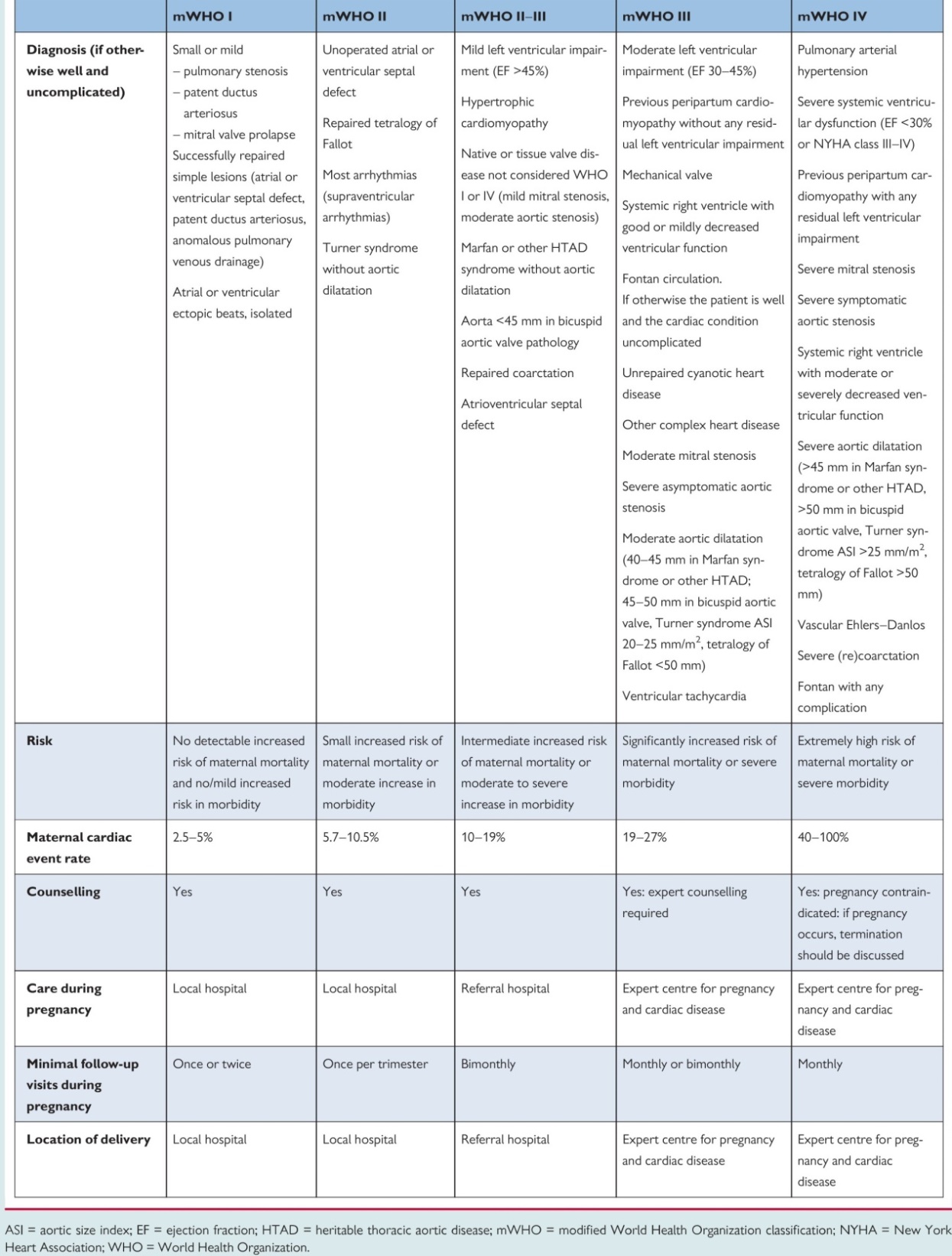

Supplement: Supplementary file 1 — Additional file 1: Supplementary Table 1. Modified World Health Organization classification of maternal cardiovascular risk. [file 12884_2022_5216_MOESM1_ESM.docx]
